# Prevalence and incidence of infections among people with type 2 diabetes: a systematic review and meta-analysis

**DOI:** 10.7189/jogh.16.04105

**Published:** 2026-03-27

**Authors:** Xue-Lei Fu, Wen-Jun Chen, Hui-Mei Zhao, Meng-Di Li, Meng-Yi Zhuang, Yang Xiao, Bei Wu, Jia Guo

**Affiliations:** 1 Xiangya School of Nursing, Central South University, Changsha, China; 2 Department of Metabolism and Endocrinology, The Second Xiangya Hospital of Central South University, Changsha, China; 3 New York University Shanghai, Shanghai, China

## Abstract

**Background:**

Although evidence suggests a greater susceptibility of type 2 diabetes to infections, the prevalence and incidence of different types of infections vary greatly, affecting clinical decision-making. We aimed to estimate the aggregate prevalence and incidence of infections among people with type 2 diabetes.

**Methods:**

We searched PubMed, Embase, Web of Science, and Cochrane Library from inception to August 2025. We combined the terms type 2 diabetes, infection, and prevalence/incidence for the search. We included studies with data on infection prevalence (percentage) and/or incidence (number of person-years at risk) among patients with type 2 diabetes. We used the quality-effects model to estimate effects and 95% confidence intervals (CIs).

**Results:**

We included 70 studies in this meta-analysis. Severe periodontitis had the highest pooled prevalence among people with type 2 diabetes (33.6%; 95% CI = 23.7–44.2), followed by skin infections (28.6%; 95% CI = 20.7–37.2) and urinary tract infections (9.7%; 95% CI = 6.5–13.5). Lower respiratory tract infections had the highest pooled incidence per 10 000 person-years (1409.2; 95% CI = 1048.1–1894.6), followed by skin infections (664.1; 95% CI = 39.4–11203.9), upper respiratory tract infections (553.6; 95% CI = 12.7–24149.2) and urinary tract infections (500.6; 95% CI = 171.3–1462.6).

**Conclusions:**

Among people with type 2 diabetes, the prevalence of severe periodontitis and the incidence of lower respiratory tract infections were the highest. We pinpointed recently prevalent high-occurrence infections in type 2 diabetes, helping to update surveillance and control priorities for diabetes care.

**Registration:**

PROSPERO: CRD42024489267

Diabetes is one of the most common and fast-growing chronic diseases worldwide [1,2]. Around 1.5 million people die of diabetes and its complications worldwide every year [3]. The complications include vascular diseases, infections, and hyperglycaemic crises. Although vascular diseases remained the largest single contributor to mortality of diabetes, the associated mortality rates were reduced by 32% every ten years due to the advanced treatment regimens for this fatal diabetes complication [2,4], along with the decline of hospitalisation rates by more than 10% from 2003 to 2018 [5]. However, the hospitalisation rates of infections among people with diabetes increased [5].

Infections are disorders caused by the invasion of the host organism [6]. Insulin deficiency and hyperglycaemia in diabetes can impair neutrophil function, antioxidant systems, and humoral immune responses, thus increasing susceptibility to infections [7]. Respiratory tract infections, urinary tract infections, and skin infections are common infections in people with diabetes [8,9]. Infections in diabetes are responsible for over USD 48 billion in the USA in 2021, accounting for about 20% of the national cost for diabetes [10]. In all, there is an increasing trend in the morbidity of infections associated with diabetes that needs to be addressed.

Given that type 2 diabetes accounts for more than 95% of diabetes cases, clarifying the epidemiology of different infections in this population is of high urgency [3]. An increasing number of individual epidemiologic studies have explored specific infections among people with type 2 diabetes, with research conducted across different countries, regions, and hospitals [11–14]. However, the prevalence and incidence of these infections vary substantially across original studies. Most existing systematic reviews and meta-analyses have focused primarily on the association between diabetes and specific infections [15–17]. Three meta-analyses quantified prevalence and incidence: one reported the prevalence of single urinary tract infections in people with diabetes [18], while two others reported the rate of type 2 diabetes among patients with specific infections [19,20].

It is therefore difficult to estimate the disease burden of diabetes-related infections, which hinders the rational allocation of prevention and control resources. To address this critical gap, a systematic review of evidence could raise awareness of prevalent ones and improve health outcomes. Therefore, we aimed to synthesise the scientific literature to estimate the aggregate prevalence and incidence of infections among people with type 2 diabetes.

## METHODS

We performed this study in accordance with the methodological guidance from the Joanna Briggs Institute [21] and reported it following the PRISMA statement [22]. We registered the review protocol on PROSPERO (CRD42024489267).

### Inclusion/exclusion criteria

Inclusion criteria for eligible studies were as follows: observational studies, with cross-sectional studies for prevalence estimation and cohort studies for incidence analysis; population-based (selecting the entire population or using probability-based sampling methods); studies with clinically confirmed infections, with confirmation sources including databases, clinical diagnoses, or medical records; and studies reported the prevalence (percentage) or incidence (number of person-years at risk) of symptomatic infections among individuals diagnosed with type 2 diabetes (or raw numbers that allowed the calculation of an estimate).

The following studies were excluded: case reports, case series, intervention studies, qualitative studies, review articles, letters, posters and conference abstracts; special populations such as patients with cancer or people who were pregnant, postoperative or immunodeficient, as their physiological status or underlying diseases independently affect infection risk; mixed diabetes populations studies with no possibility of identifying the type 2 diabetes subpopulation; studies reported prevalence and incidence of parasitosis, given these infections are strongly linked to specific environmental exposures; studies on infections transmitted solely via vector-borne, blood/body fluid, sexual contact, or mother-to-child vertical routes, as these infections are driven primarily by exposure to specific transmission pathways; and studies on large-scale communicable diseases (*e.g.* severe acute respiratory syndrome, COVID-19), due to the population-wide universal susceptibility characteristic of these diseases.

### Retrieval of studies

We searched for scientific literature published in PubMed, Embase, Web of Science, and Cochrane Library, supplemented by searching the grey literature on relevant websites (*e.g.* Centers for Disease Control and Prevention) and Google Scholar from inception to August 2023, with updated searches conducted in August 2025 (Table S1 in the **Online Supplementary Document**). Keywords were various combinations of terms for type 2 diabetes and various infections, as well as their prevalence or incidence. To ensure comprehensive coverage of all possible infections, we determined the infection-related search terms primarily by referencing the term ‘Infections’ and its subcategories under the Diseases Category of the Medical Subject Headings system, and secondarily by reviewing relevant reviews focusing on diabetes-related infections. We included only studies that targeted humans. We also manually searched the reference lists of identified systematic reviews and included articles to ensure the completeness of the collected articles. We exported the retrieved records to an Endnote library (version X9) and filtered the duplicates. Two reviewers (FX and ZH) independently screened titles and abstracts against the eligibility criteria, followed by a full-text review of all potentially eligible studies. Any disagreements were resolved *via* consensus discussions.

### Data extraction

Two independent reviewers (LM and ZM) extracted the data using two standardised data collection tables, one for prevalence and one for incidence studies. A third author (FX) checked the accuracy and integrity of the information. Key study characteristics included first author, year of publication, study period, country, data source, demographic information (age, gender, and diabetes duration), types of infections, number of cases, and sample size. We extracted the number of people with various infections along with the study sample size of type 2 diabetes for calculating the prevalence. We identified the number of new infections and recorded the person-years risk to calculate the incidence.

### Quality assessment

Two reviewers (FX and ZH) independently assessed the quality of included studies using the Joanna Briggs Institute’s Critical Appraisal Checklist for Studies Reporting Prevalence Data [21]. The tool consists of nine items that could be rated as ‘yes’, ‘no’, ‘unclear’ or ‘not applicable’. We categorised each study as either low, moderate, or high quality based on the total score [23].

### Data analysis

We used Meta-XL, version 5.3 (EpiGear International Pty Ltd, Sunrise Beach, Queensland, Australia) to conduct a meta-analysis to determine the pooled prevalence and incidence of various infections. When the number of included studies was adequate (n >2), we conducted meta-analyses. We initially intended to employ a random-effects meta-analysis. However, given the over-dispersed and highly heterogeneous nature of the data, this model would have underestimated statistical error and produced spuriously overconfident estimates [24,25]. Thus, we used a quality-effects model, assigning higher weights to higher-quality studies, which is more robust and maintains the correct coverage probability of the confidence interval (CI), regardless of the level of heterogeneity [26,27]. We used a double arcsine transformation to stabilise the variance and avoid overweighting studies with values near 0% or 100% [28]. We performed subgroup analyses by continent and diagnostic criteria to explore sources of heterogeneity. We conducted meta-analyses for gender when data were available. We did not perform tests for publication bias (*e.g.* Egger’s test, Begg’s test, and funnel plots) because the assumption that positive results are more often published is not necessarily true for proportional studies [29].

## RESULTS

We included 70 studies (Figure S1 in the **Online Supplementary Document**), of which 37 reported prevalence [11–13,30–63], and 34 reported incidence [14,39,64–95]. For prevalence-focused studies, the sample size ranged from 40 to 293 124 persons (**Table 1**). Among these studies, ten were categorised as high quality, 22 as moderate quality, and five as low quality (Table S2 in the **Online Supplementary Document**). For incidence-focused studies, all studies were categorised as either moderate quality or high quality (Table S3 in the **Online Supplementary Document**), and the sample size for incidence ranged from 80 to 5 928 052 persons, with a mean follow-up time spanning 1–12 years (**Table 2**).

**Table 1:** Characteristics of included studies for the prevalence of infections among people with type 2 diabetes

Author, year	Study period	Country	Data source	Male, %	Age in years, x̄ (SD)	DM duration in years, x̄ (SD)	Infection type	Number of cases	Sample size	Prevalence, %
Sorescu *et al.*, 2024 [63]	2023	Romania	Clinical diagnosis	NR	NR	NR	UTI	225	1142	19.7
Liu *et al.*, 2023 [62]	2017	China	Clinical diagnosis	38.4	NR	NR	PTB	160	89 549	0.18
Todescan *et al.*, 2023 [59]	2013–2015	Canada	Clinical diagnosis	34.7	14.5 (2.2)	2.6 (2.1)	Periodontitis (mild, moderate, severe)	55 (10, 36, 9)	121	45.5 (8.3, 29.8, 7.4)
Al Qurabiy *et al.*, 2022 [31]	2021	Iraq	Clinical diagnosis	NR	NR	NR	UTI	61	93	65.6
Fu *et al.*, 2021 [40]	2007–2014	China	Medical records	55.7	65.1 (15.2)	7.3 (7.3)	PTB	47	9750	0.5
Wachinou *et al.*, 2021 [61]	2015–2017	Benin, Guinea, Senegal	Clinical diagnosis	NR	NR	NR	PTB	106	5375	2.0
Carrondo *et al.*, 2020 [34]	2015	Portugal	Database	51.1	NR	NR	UTI	1190	7347	16.2
Dabhi *et al.*, 2020 [36]	2014–2015	India	Clinical diagnosis	57.6	NR	7.2	PTB	53	1000	5.3
Ekeke *et al.*, 2020 [37]	2018	Nigeria	Clinical diagnosis	NR	NR	NR	PTB	18	3328	0.5
Rasid *et al.*, 2020 [53]	2019	Malaysia	Clinical diagnosis	52.0	66.0 (13.0)	10.0 (10.0)	Skin infection	94	271	34.7
Saleh *et al.*, 2020 [55]	2015–2020	USA	Database	48.9	NR	NR	Periapical abscess	766	52 493	1.5
He *et al.*, 2018 [43]	2013–2016	China	Clinical diagnosis	50.7	59.3 (14.1)	6.3 (0.5)	UTI	409	3652	11.2
Lin *et al.*, 2018 [12]	1998–2010	China	Database	51.1	50.7 (12.4)	NR	PTB	917	49 028	1.9
Borowczyk *et al.*, 2017 [33]	NR	Poland	Clinical diagnosis	0.0	NR	NR	UTI	15	40	37.5
Nitta *et al.*, 2017 [49]	NR	Japan	Clinical diagnosis	61.1	53.8 (9.9)	9.6 (7.7)	Periodontitis (severe)	406 (211)	620	65.5 (34.0)
Pham *et al.*, 2018 [50]	2015	Vietnam	Clinical diagnosis	NR	NR	NR	Periodontitis	109	215	50.7
Pumerantz *et al.*, 2017 [52]	2014–2016	UK, USA	Clinical diagnosis	49.4	53.6 (11.0)	NR	Periodontitis (severe)	128 (57)	253	50.6 (22.5)
Chiţă *et al.*, 2017 [35]	2011–2012	Romania	Clinical diagnosis	NR	NR	NR	UTI	274	2167	12.6
Masood *et al.*, 2016 [48]	2015	Pakistan	Clinical diagnosis	NR	NR	NR	PTB	25	227	11.0
Hamdan *et al.*, 2015 [42]	2013	Sudan	Clinical diagnosis	NR	NR	NR	UTI	35	193	18.1
Lin *et al.*, 2015 [45]	2012	China	Clinical diagnosis	48.0	74.2 (5.9)	NR	PTB	12	3087	0.4
Fu *et al.*, 2014 [39]	2010	USA	Database	50.7	56.0 (13.4)	NR	UTI	8456	89 790	9.4
Masi *et al.*, 2014 [47]	2007–2009	UK	Clinical diagnosis	NR	NR	NR	Gingivitis	104	371	28.0
							Periodontitis (mild, moderate, severe)	223 (0, 69, 154)	371	60.1 (0.0, 18.6, 41.5)
Yu *et al.*, 2014 [11]	2008–2011	USA	Database	52.1	NR	NR	UTI	6014	73 151	8.2
Al-Rubeaan *et al.*, 2013 [32]	1993–2009	Saudi Arabia	Clinical diagnosis	NR	NR	NR	UTI	213	831	25.6
Susanto *et al.*, 2011 [58]	2008–2009	Indonesia	Clinical diagnosis	44.9	56.7 (9.4)	NR	Periodontitis	72	78	92.3
Preshaw *et al.*, 2010 [51]	NR	Sri Lanka	Clinical diagnosis	48.8	45.7 (12.7)	NR	Gingivitis	81	285	28.4
							Periodontitis	95	285	33.3
Silva *et al.*, 2010 [57]	2006	Brazil	Clinical diagnosis	NR	NR	NR	Periodontitis	46	212	21.7
Ahmed *et al.*, 2009 [30]	2008	Pakistan	Clinical diagnosis	NR	NR	NR	Skin infection	105	320	32.8
Fernandes *et al.*, 2009 [38]	2004–2006	USA	Clinical diagnosis	26.4	NR	10.6 (9.9)	Periodontitis (mild, moderate, severe)	235 (2, 166, 67)	235	100.0 (0.9, 70.6, 28.5)
Hintao *et al.*, 2007 [44]	NR	Thailand	Clinical diagnosis	49.5	54.3 (8.7)	8.7 (5.7)	Periodontitis (severe)	103 (89)	105	98.1 (84.8)
Malazy *et al.*, 2007 [46]	2002–2005	Iran	Clinical diagnosis	NR	NR	NR	Vaginitis	106	151	70.2
Movahed *et al.*, 2007 [13]	1990–2000	USA	Medical records	97.8	65.8 (11.3)	NR	IE	1340	293 124	0.5
Sasmaz *et al.*, 2004 [56]	NR	Turkey	Clinical diagnosis	29.8	54.0 (17.0)	11.0 (4.0)	Skin infection	48	151	31.8
Tsai *et al.*, 2002 [60]	1988–1994	USA	Database	50.4	NR	NR	Severe periodontitis	303	4343	7.0
Goswami *et al.*, 2001 [41]	1998–1999	India	Clinical diagnosis	NR	NR	5.0 (3.4)	UTI	6	53	11.3
Romano *et al.*, 1998 [54]	NR	Italy	Clinical diagnosis	43.3	59.0 (16.0)	NR	Skin infection	81	393	20.6

AAP – American Academy of Periodontology, CDC – Centers for Disease Control and Prevention, CPI – Community Periodontal Index, DM – diabetes mellitus, EWP – European Workshop of Periodontology, ICD – International Classification of Disease, IE – infective endocarditis, NR – not reported, PTB – pulmonary tuberculosis, SD – standard deviation, UTI – urinary tract infection, WHO - World Health Organization, x̄ – mean

**Table 2:** Characteristics of included studies for the incidence of infections among people with type 2 diabetes

Author, year	Study period	Country	Data source	Diagnostic criteria	Sample size	Male, %	Age in years, x̄ (SD)	DM duration in years, x̄ (SD)	Infection type	Number of cases	Person-year	Incidence rate (/10 000 person-years)
Kim *et al.*, 2025 [77]	2009–2012	Korea	Database	ICD-10	1 762 108	60.9	59.0 (11.7)	NR	IE	828	8 882 310	0.9
Lee *et al.*, 2025 [80]	2009–2020	Korea	Database	ICD-10	297 445	63.3	56.6 (12.1)	NR	Sepsis	15 199	2 879 391	52.8
Feleke *et al.*, 2024 [69]	2010–2019	Australia	Database	ICD-10	NR	NR	NR	NR	Gastrointestinal tract infections	43 045	7 540 919	57.1
									UTI	54 076	7 540 919	71.7
									Sepsis	43 774	7 540 919	58.0
									Pneumonia	74 697	7 540 919	99.1
									Osteomyelitis	15 756	7 540 919	20.9
									Cellulitis	63 753	7 540 919	84.5
Chien *et al.*, 2023 [67]	2000–2015	China	Database	ICD-9	157 798	NR	NR	NR	Periodontitis	1662	1 599 752	10.4
Antonio-Arques *et al.*, 2022 [64]	2007–2016	Spain	Database	Spanish Consensus	8004	61.2	57.7 (14.2)	3.2 (5.8)	PTB	48	68 605	7.0
Lopez-de-Andres *et al.*, 2022 [82]	2016–2020	Spain	Database	ICD-10	NR	NR	NR	NR	IE	2668	14 899 474	1.8
Poirrier *et al.*, 2022 [89]	2012–2018	USA	Databases	ICD-9	5 928 052	NR	NR	NR	HZ	124 683	12 748 009	97.8
Wang *et al.*, 2021 [93]	2000–2009	China	Database	ICD-9	44 728	54.2	55.6 (13.9)	NR	PLA	166	282 924	5.9
Wu *et al.*, 2021 [95]	1997–2013	China	Database	ICD-9	67 852	53.6	NR	NR	Peritonsillar abscess	61	493 627	1.2
Li *et al.*, 2020 [81]	2004–2015	China	Database	NTP Guidelines	240 692	45.8	NR	NR	PTB	439	855 782	5.1
Pan *et al.*, 2020 [87]	2001–2013	China	Database	ICD-9	45 339	54.0	57.7 (13.7)	NR	PTB	539	240 782	22.4
									HZ	1974	233 714	84.5
									PLA	237	240 034	9.9
Banerjee *et al.*, 2019 [65]	NR	India	Clinical diagnosis		80	46.3	54.9 (11.4)	9.7 (5.6)	UTI	10	80	1250.0
de Miguel-Yanes *et al.*, 2019 [68]	2001–2015	Spain	Database	ICD-9	NR	NR	NR	NR	IE	3436	34 210 606	1.0
Ko *et al.*, 2019 [78]	2000–2010	China	Database	ICD-9	613 921	48.0	60.1 (12.7)	NR	PLA	5336	4 623 916	11.5
Carey *et al.*, 2018 [14]	2008–2015	UK	Database	ICD-10	96 630	55.3	NR	NR	Bone and joint infections	1071	473 894	22.6
									Acute Cholecystitis	1035	514 925	20.1
									IE	100	500 000	2.0
									Gastrointestinal tract infections	3930	497 468	79.0
									Otitis externa	7091	500 071	141.8
									LRTI (Pneumonia, PTB)	58 667 (7935, 123)	500 534 (496 869, 492 000)	1172.1 (159.7, 2.5)
									Meningitis	37	528 571	0.7
									Sepsis	2612	493 762	52.9
									Skin infection (Cellulitis)	43 312 (18 974)	494 759 (494 759)	875.4 (383.5)
									URTI (Acute sinusitis)	32 448 (6605)	501 708 (500 000)	646.8 (132.1)
									UTI	28705	499 217	575.0
Ferreira *et al.*, 2018 [70]	2014–2015	UK	Database	Read-Code	160 760	NR	NR	NR	Hepatitis B	28	689 655	0.4
Tseng, 2018 [91]	1999–2005	China	Database	ICD-9	164 267	53.7	61.9 (10.1)	9.6 (2.2)	PTB	2336	768 839	30.4
Wang *et al.*, 2018 [92]	2000–2010	China	Database	ICD-9	404 971	55.2	56.2 (12.4)	NR	PLA	1980	2 059 668	9.6
Nichols *et al.*, 2017 [86]	2006–2012	USA	Database	ICD-9	39 295	52.2	60.2 (12.8)	3.4 (4.6)	UTI	13 085	216 123	605.4
									Genital Infection	7898	216 123	365.4
Qiu *et al.*, 2017 [90]	2004–2009	China	Database	NTP Guidelines	170 399	41.1	59.3 (11.2)	NR	PTB	785	654 977	12.0
Mor *et al.*, 2016 [84]	2004–2012	Denmark	Database	ICD-8	155 158	55.0	65.6 (13.6)	NR	URTI	1631	728 125	22.4
									Infection of heart and blood vessels	282	742 105	3.8
									Pneumonia	10 720	709 464	151.1
									Gastrointestinal tract infections	2578	726 197	35.5
									UTI (Emphysematous cystitis, Emphysematous pyelonephritis)	8093 (610, 588)	735 000 (734 940, 735 000)	110.1 (8.3, 8.0)
									Infection of the central nervous system	312	725 581	4.3
									Perirenal abscess	78	709 091	1.1
									Intraabdominal infection	4356	720 000	60.5
									Skin infection	5637	717 176	78.6
									Septicemia	4021	728 442	55.2
									PTB	112	746 667	1.5
									Emphysematous cholecystitis	597	728 049	8.2
Heo *et al.*, 2015 [73]	2009	Korea	Database	ICD-10	331 601	52.8	56.8 (13.1)	NR	PTB	1533	831 486	18.4
Pealing *et al.*, 2015 [88]	1990–2013	UK	Database	Read code	216 545	NR	NR	NR	PTB	189	1 138 000	1.7
Wilke *et al.*, 2015 [94]	2010–2012	Germany	Database	ICD-10	456 586	43.9	72.8	NR	UTI	82 439	944 318	873.0
Fu *et al.*, 2014 [39]	2010	USA	Database	ICD-9	82 239	NR	NR	NR	UTI	5896	82 239	716.9
Guignard *et al.*, 2014 [71]	1997–2006	USA	Database	ICD-9	380 401	58.8	NR	NR	HZ	6475	1 410 654	45.9
Kang *et al.*, 2014 [76]	2007-2010	Korea	Database	ICD-10	840 899	59.1	56.3 (13.4)	NR	PTB	4075	1 626 376	25.1
McDonald *et al.*, 2014 [83]	1997–2011	UK	Database	Read code	218 805	NR	NR	NR	LRTI (Pneumonia)	139 301 (9697)	912 253 (941 456)	1527.0 (103.0)
									UTI	91 574	919 418	996.0
									Sepsis	2461	980 478	25.1
Hamilton *et al.*, 2013 [72]	1993–2010	Australia	Database	ICD-9/10	1294	48.8	64.1 (11.3)	NR	Pneumonia	181	15 535	116.5
									Cellulitis	107	15 535	68.9
									Osteomyelitis	19	15 535	12.2
Kuo *et al.*, 2013 [79]	2000–2011	China	Database	ICD-9	63 730	51.0	55.5 (12.6)	NR	PTB	1022	395 242	25.9
Hirji *et al.*, 2012 [74]	NR	UK	Database	OXMIS/ Read codes	62 537	0.0	64.1 (14.4)	NR	Vaginitis	1243	59 279	209.7
					73 383	100.0	61.4 (12.6)	NR	Balanitis	592	70 088	84.5
Hirji *et al.*, 2012 [75]	1990–2007	UK	Database	OXMIS/ Read code	135 920	54.0	62.6 (13.5)	NR	UTI	5967	127 228	469.0
Muller *et al.*, 2005 [85]	2000–2002	Netherlands	Clinical diagnosis	ICPC code	6712	46.1	65.7 (12.7)	NR	URTI (Acute rhinolaryngitis, Acute sinusitis, Acute tonsillitis, Streptococcal angina)	495 (346, 132, 15, 2)	6712	737.5 (515.5, 196.7, 22.3, 3.0)
									Otitis media	19	6712	28.3
									LRTI (Acute bronchitis, Pneumonia)	344 (223, 106)	6712	512.5 (332.2, 157.9)
									Pleuritis	1	6712	1.5
									UTI (Cystitis, Acute pyelonephritis)	451 (442, 6)	6712	671.9 (658.5, 8.9)
									Prostatitis	16	6712	23.8
									Skin infection (Cellulitis, Impetigo)	508 (47, 11)	6712	756.9 (70.0, 16.4)
									Otitis externa	137	6712	204.1
Bartelink *et al.*, 1998 [66]	NR	Netherlands	Diagnosis or prescription drug records	328	37.2	69.7 (10.5)	NR	NR	Conjunctivitis	26	656	396.3
									LRTI	40	656	609.8
									Otitis externa	20	656	304.9
									URTI	77	656	1173.8
									UTI	87	656	1326.2

DM – diabetes mellitus, HZ – herpes zoster, IE – infective endocarditis, ICPC – International Classification of Primary Care, LRTI – lower respiratory tract infections, NR – not reported, NTP – National TB Control Program, PLA – pyogenic liver abscess, PTB – pulmonary tuberculosis, SD – standard deviation, URTI – upper respiratory tract infections, UTI – urinary tract infections, x̄ – mean

### Prevalence of infections among people with type 2 diabetes

#### Oral infections

A total of 11 studies reported the prevalence of periodontitis [38,44,47,49–52,57–60], with reported prevalence rates ranging from 21.7% to 100%. Given the substantial heterogeneity in the diagnostic criteria for periodontitis, we did not perform data pooling for the meta-analysis. Among these studies, two adopted the World Health Organization’s Community Periodontal Index Codes 3/4/X as the diagnostic criteria, and their reported prevalence rates were 60.1% [47] and 65.5% [49]. Another two employed the criteria established by the Centers for Disease Control and Prevention and the American Academy of Periodontology, and their prevalence rates were 45.5% [59] and 50.7% [50]. Additionally, three studies defined severe periodontitis using the criterion of ‘pocket-probing depth ≥6mm’ [47,49,52], and the pooled prevalence rate was 33.6% (95% CI = 23.7–44.2; *I*^2^ = 92%) (**Table 3**). Although the diagnostic criteria for gingivitis were inconsistent between the two studies, the reported prevalence rates were both around 28% [47,51]. By contrast, the reported prevalence of periapical abscess was considerably lower at 1.5% [55].

**Table 3:** Pooled prevalence and incidence of infections among people with type 2 diabetes*

	**Studies, n**	**Rate, % (95% CI)**	***I*^2^, %**
**Prevalence of infections**			
Severe periodontitis	3	33.6 (23.7–44.2)	92.1
Urinary tract infections	11	9.7 (6.5–13.5)	98.9
Skin infections	4	28.6 (20.7–37.2)	86.1
Pulmonary tuberculosis	8	0.9 (0.0–2.3)	99.5
**Incidence of infections†**			
Upper respiratory tract infections	4	553.6 (12.7–24 149.2)	100.0
Lower respiratory tract infections	4	1409.2 (1048.1–1894.6)	99.9
Pneumonia	6	107.8 (74.6–155.9)	99.8
Pulmonary tuberculosis	11	20.1 (10.8–37.6)	99.8
Urinary tract infections	11	500.6 (171.3–1462.6)	100.0
Skin infections	3	664.1 (39.4–11 203.9)	100.0
Cellulitis	4	119.5 (22.4–638.3)	100.0
Herpes zoster	3	94.3 (39.7–224.4)	99.9
Sepsis	4	54.7 (39.2–76.4)	99.8
Gastrointestinal tract infections	3	57.1 (34.7–94.1)	99.8
Other			
*Otitis externa*	3	143.1 (80.8–253.4)	93.2
*Infectious endocarditis*	4	1.3 (0.8–2.0)	99.5
*Pyogenic liver abscess*	4	10.8 (8.3–14.0)	97.4

CI – confidence interval

*Values are presented as % (95% CI) unless specified otherwise.

†The rate is presented as a number per 10 000 person-years.

#### Urinary tract infections

The prevalence of urinary tract infection was reported in 11 studies [11,31–35,39,41–43,63], with three using International Classification of Disease, 9th Revision (ICD-9) diagnostic codes, and the remaining eight confirming cases via urine culture. The pooled prevalence of urinary tract infections was 9.7% (95% CI = 6.5–13.5; *I*^2^ = 99%) (**Table 3**). Subgroup analyses by diagnostic criteria showed a pooled prevalence of 9.3% (95% CI = 6.8–12.0; *I*^2^ = 100%) in the ICD-9 subgroup and 14.7% (95% CI = 7.1–23.2; I^2^ = 97%) in the urine culture subgroup (Figure S2 in the **Online Supplementary Document**). The pooled prevalence estimate of urinary tract infections among females was 13.0% (95% CI = 2.7–28.4; n = 3; *I*^2^ = 98%) (Figure S3 in the **Online Supplementary Document**). The prevalence of urinary tract infections was 14.1% (95% CI = 0.0–38.2; n = 4; *I*^2^ = 99%) in Asia and 16.2% (95% CI = 11.7–21.1; n = 4; *I*^2^ = 93%) in Europe (Figure S4 in the **Online Supplementary Document**).

#### Skin infections

Four studies reported the prevalence of skin infections defined as clinically diagnosed bacterial, fungal, and viral infections [30,53,54,56]. Skin infections had a combined prevalence estimate of 28.6% (95% CI = 20.7–37.2; *I*^2^ = 86%) (**Table 3**).

#### Pulmonary tuberculosis

Eight studies reported the prevalence of pulmonary tuberculosis, with a pooled prevalence estimate of 0.9% (95% CI = 0.0–2.3; *I*^2^ = 100%) (**Table 3**) [12,36,37,40,45,48,61,62]. Of these studies, six confirmed cases by positive sputum culture, smear microscopy, or physician diagnosis based on clinical-epidemiological data and radiographic abnormalities, with a corresponding pooled prevalence of 0.3% (95% CI = 0.0–2.1; *I*^2^ = 99%); the remaining two used ICD-9 diagnostic codes for case confirmation (Figure S5 in the **Online Supplementary Document**). The prevalence of pulmonary tuberculosis in Asia was 0.8% (95% CI = 0.0–2.5; n = 6; *I*^2^ = 100%) (Figure S6 in the **Online Supplementary Document**).

#### Other infections

Since only one study reported on certain diseases, its data cannot be combined with those from other studies. Although only one study reported the prevalence of vaginitis, the rate was remarkably high, reaching 70.2% [46]. In contrast, the prevalence of infective endocarditis was relatively low, at 0.5% [13] (**Table 1**).

### Incidence of infections among people with type 2 diabetes

#### Upper respiratory tract infections

The four studies on upper respiratory tract infection were identified based on diagnosis or prescription drug records, as well as the International Classification of Diseases, 8th Revision (ICD-8), 10th Revision (ICD-10), and International Classification of Primary Care (ICPC) coding systems [14,66,84,85]. The pooled incidence rate of upper respiratory tract infections was 553.6 per 10 000 person-years (95% CI = 12.7–24 149.2; *I*^2^ = 100%) (**Table 3**).

Incidence rates of acute sinusitis per 10 000 person-years were 132.1 when identified using ICD-10 and 196.7 when identified using ICPC [14,85]. Reported incidence rates per 10 000 person-years were 515.5 for acute rhinolaryngitis and 22.3 for acute tonsillitis [85], and 1.2 for peritonsillar abscess [95].

#### Lower respiratory tract infections

The four studies on lower respiratory tract infection were based on confirmed case records, namely diagnosis or prescription drug records, ICPC codes, Read codes, and ICD-10 [14,66,83,85]. Lower respiratory tract infections had a pooled incidence of 1409.2 (95% CI = 1048.1–1894.6; *I*^2^ = 100%) per 10 000 person-years among people with type 2 diabetes (**Table 3**).

Six studies reported pneumonia incidence [14,69,72,83–85], with a pooled incidence rate of 107.8 (95% CI = 74.6–155.9; *I*^2^ = 100%) per 10 000 person-years. One study used ICPC codes, another Read codes, and four the ICD system. Studies using the ICD system indicated an overall incidence of 108.3 (95% CI = 66.7–176.0; *I*^2^ = 100%) per 10 000 person-years (Figure S7 in the **Online Supplementary Document**). The incidence rate of pneumonia was 134.7 (95% CI = 102.8–176.5; n = 4; *I*^2^ = 100%) per 10 000 person-years in Europe (Figure S8 in the **Online Supplementary Document**). In comparison, the incidence of acute bronchitis was higher, at 332.2 per 10 000 person-years [85].

Pulmonary tuberculosis incidence was identified based on the Spanish Consensus, National Tuberculosis Plan Guidelines, ICD-8/9/10, or Read code diagnostic criteria [14,64,73,76,79,81,84,87,88,90,91]. The pooled incidence rate of pulmonary tuberculosis was 20.1 per 10 000 person-years (95% CI = 10.8–37.6; n = 11; *I*^2^ = 100%) (**Table 3**). Studies with the ICD diagnostic system indicated an overall incidence of 23.4 per 10 000 person-years (95% CI = 14.1–38.9; n = 7; *I*^2^ = 100%) (Figure S9 in the **Online Supplementary Document**). The incidence of pulmonary tuberculosis per 10 000 person-years was 22.3 (95% CI = 14.7–33.8; n = 7; *I*^2^ = 100%) in Asia and 2.1 (95% CI = 1.2–3.7; n = 4; *I*^2^ = 97%) in Europe (Figure S10 in the **Online Supplementary Document**).

#### Urinary tract infections

Among the 11 studies investigating the incidence of urinary tract infection, six used ICD coding systems as diagnostic criteria, and the remaining five each used a distinct criterion, namely urine culture, diagnosis or prescription drug records, ICPC code, OXMIS/Read code, or Read code [14,39,65,66,69,75,83–86,94]. The pooled incidence rate of urinary tract infections was 500.6 per 10 000 person-years (95% CI = 171.3–1462.6; *I*^2^ = 100%) (**Table 3**). Studies with the ICD diagnostic system indicated an overall incidence of 360.9 per 10 000 person-years (95% CI = 91.1–1429.8; n = 6; *I*^2^ = 100%) (Figure S11 in the **Online Supplementary Document**). Incidence of urinary tract infections per 10 000 person-years was higher in females at 1379.0 (95% CI = 627.8–3029.1; n = 3; *I*^2^ = 100%) compared to 502.9 (95% CI = 430.6–587.4; n = 3; *I*^2^ = 97%) in males (Figure S12 in the **Online Supplementary Document**). The incidence rate was 795.1 (95% CI = 428.3–1475.8; n = 7; *I*^2^ = 100%) per 10 000 person-years in Europe (Figure S13 in the **Online Supplementary Document**). The incidence of cystitis was reported at 658.5 per 10 000 person-years [85], whereas the rates of acute pyelonephritis, emphysematous cystitis, and emphysematous pyelonephritis all remained around 8.0 per 10 000 person-years [84,85].

#### Skin infections

Three studies investigated skin infections using ICD-8, ICD-10, and ICPC codes [14,84,85], with a pooled incidence rate of 664.1 per 10 000 person-years (95% CI = 39.4–11 203.9; *I*^2^ = 100%). The pooled incidence rate of cellulitis per 10 000 person-years was 119.5 (95% CI = 22.4–638.3; n = 4; *I*^2^ = 100%), while it was 119.5 (95% CI = 22.3–640.3; n = 3; *I*^2^ = 100%) for ICD-diagnosed cellulitis [14,69,72,85] (Figure S14 in the **Online Supplementary Document**). Herpes zoster was identified using ICD-9 codes, with an incidence rate of 94.3 per 10 000 person-years (95% CI = 39.7–224.4; n = 3; *I*^2^ = 100%) (**Table 3**) [71,87,89]. The incidence of impetigo was 16.4 per 10 000 person-years [85].

#### Other infections

Sepsis had the pooled incidence rate of 54.7 per 10 000 person-years (95% CI = 39.2–76.4; n = 4; *I*^2^ = 100%), based on confirmed diagnostic codes [14,69,80,83]. Gastrointestinal tract infections, confirmed by ICD codes, had a pooled incidence rate of 57.1 per 10 000 person-years (95% CI = 34.7–94.1; n = 3; *I*^2^ = 100%) (**Table 3**) [14,69,84]. The incidence of genital infections was 365.4 per 10 000 person-years [83], with a notable rate of 209.7 per 10 000 person-years observed for vaginitis [74].

Compared with the otitis media incidence rate of 28.3 per 10 000 person-years [85], otitis externa had a significantly higher incidence rate at 143.1 (95% CI = 80.8–253.4; n = 3; *I*^2^ = 93%), identified from diagnosis/prescription drug records, ICD-10 and ICPC codes [14,66,85]. Conjunctivitis also presented a high incidence rate of 396.3 per 10 000 person-years [66], while the incidence rate of periodontitis was low (10.4 per 10 000 person-years) [67]. Also identified with ICD codes, pooled incidence rates were 1.3 (95% CI = 0.8–2.0; n = 4; *I*^2^ = 100%) for infectious endocarditis [14,68,77,82] and 10.8 (95% CI = 8.3–14.0; n = 4; *I*^2^ = 97%) for pyogenic liver abscess [78,87,92,93]. We also reported the incidence rates per 10 000 person-years of other infections that could not be pooled (**Table 2**).

## DISCUSSION

We found that among infections in this population, severe periodontitis had the highest prevalence at 33.6%, while lower respiratory tract infections had the highest incidence, at 1409.2 per 10 000 person-years. Overall, these findings provide new insights into the high burden of infections, particularly periodontitis, respiratory tract infections, skin infections, and urinary tract infections, associated with type 2 diabetes, and highlight crucial areas for future research and clinical practice.

We found that the prevalence of severe periodontitis was higher than that of gingivitis among people with type 2 diabetes. Because all cases of chronic periodontitis originate from untreated gingivitis, our findings suggest that many individuals do not seek dental care until gingivitis progresses to periodontitis, thereby missing the window for early intervention [96]. This delay likely reflects limited public and professional awareness – both patients and, in some cases, healthcare providers may underestimate the importance of gingival inflammation and fail to recognise the bidirectional relationship between poor oral health and diabetes [97,98]. Early detection and timely periodontal care are therefore essential, as controlling gingivitis can halt progression to periodontitis and may also contribute to better glycaemic control in people with diabetes. Additionally, the incidence of periodontitis was relatively low (10.4 per 10 000 person-years) despite its high prevalence. This discrepancy likely reflects the chronic and slowly progressive nature of periodontitis: most cases develop over years and therefore accumulate in prevalence, whereas relatively few new cases arise in any single year. Additionally, improvements in population-level oral health behaviours, increased use of dental services, and declines in smoking rates in many regions may have reduced the incidence of new-onset disease [99].

Lower respiratory tract infections had the leading incidence of infections (1409.2 per 10 000 person-years), which was higher than the rate of 629.5 per 10 000 persons among the general population in 2019 [100]. This cannot be attributed to the COVID-19 pandemic, as the included studies on lower respiratory infections were not conducted during the COVID-19 period. The incidence rate of upper respiratory tract infections was only 553.6 per 10 000 person-years. This surprising disparity between upper and lower respiratory tract infections may be due to people often managing the symptoms of upper respiratory tract infections with home remedies and over-the-counter medications [84,101]. They tend to seek medical help only when their symptoms worsen progressively, and they are unable to cope [101], leading to a serious underestimate of reporting upper respiratory infections. Due to the lack of relevant data, we could not determine the prevalence of respiratory tract infections in patients with type 2 diabetes.

In this study, skin infections ranked second in both prevalence and incidence. Among people with type 2 diabetes, the prevalence of skin infections was 28.6%, while the incidence was 664.1 per 10 000 person-years. Our findings were much higher than those of general hospitalised patients, with a prevalence of 16.5 cases per 1000 hospitalised patients (16.0 in 2014, 17.4 in 2025, and 15.5 in 2016) [102]. In addition to significant metabolic and immunological alterations caused by hyperglycaemia, the higher skin pH in people with diabetes also facilitates bacterial and fungal colonisation, increasing susceptibility to skin infections [103,104].

Previous evidence suggests that urinary tract infections are the most common bacterial infections among diabetic patients [105], and we also found high prevalence and incidence of urinary tract infections among people with type 2 diabetes. This could be due to various reasons. First, urinary glucose elevation may promote pathogenic bacterial growth. Second, impaired humoral, cellular, and innate immunity in diabetic patients also contributes to the pathogenesis of urinary tract infections. Additionally, genitourinary autonomic neuropathy causes voiding dysfunction and urinary retention, reducing bacterial clearance through urination and further facilitating bacterial growth [106]. Furthermore, we also found a higher prevalence and incidence of urinary tract infections among female patients with type 2 diabetes compared to males, aligning with non-diabetic epidemiological patterns [107].

### Strengths and limitations

The epidemiological data we obtained through this systematic review and meta-analysis are the most comprehensive globally regarding the prevalence and incidence of infections in type 2 diabetes. This offers a more accurate description of the epidemiology of various infections than that provided by single studies. Additionally, while nearly all included studies on infection incidence attained high methodological quality, those focusing on infection prevalence exhibited substantial heterogeneity in quality, particularly for skin infection prevalence. A key methodological strength of our analysis is the proactive adoption of a rigorous quality-effects model to mitigate the impact of inter-study quality heterogeneity on the reliability of our pooled estimates [26,27].

However, our study has several limitations. First, consistent with most prevalence-focused meta-analyses, we observed high heterogeneity. We performed subgroup analyses to explore heterogeneity by continent and diagnostic criteria for infection, yet neither factor was identified. We could not conduct subgroup analyses for other variables (*e.g.* sex, data source, drug use, socioeconomic status, race/ethnicity, and blood glucose levels) due to the limited number of included studies. Second, although diabetes type is generally coded in electronic records, there are risks of incorrect or inadequate classification [108]. To address this, some studies have opted to further categorise controversial diabetes cases by patients' age and medical management, or to exclude cases with a high likelihood of misclassification [14,84,85], which may introduce bias. Furthermore, restricting literature searches to English-language databases and over-relying on electronic health record-based studies may omit research from non-English regions and settings with underdeveloped digital health infrastructure. This selection bias compromises the generalisability of our findings. Notably, existing research on infections among people with type 2 diabetes remains insufficient, highlighting an urgent need to expand research scope and increase the number of studies for systematic investigation.

### Implications

Our findings shed light on the high burden of infections among patients with diabetes, providing actionable implications for clinical practice and public health interventions. For clinicians, beyond standard diabetes education, modules on reducing infection risk should be incorporated into both initial diagnosis consultations and routine follow-up visits. Interdisciplinary collaboration between dental practitioners and endocrinologists is further recommended, with annual periodontal assessments integrated into regular diabetes follow-up protocols. For public health practitioners, proactive infection-prevention plans should be developed for people with diabetes. Community health service centres may prioritise vaccination recommendations and respiratory protection guidance for people with diabetes during influenza seasons.

## CONCLUSIONS

The pooled prevalence of severe periodontitis and the incidence of lower respiratory tract infections were the highest among people with type 2 diabetes; thus, medical staff and patients should prioritise both for proactive prevention. Beyond these key priorities, other common infections in this patient group also merit targeted attention, including those affecting the gingival tissues, upper respiratory tract, urinary tract, genitals, and skin.

## Additional material


Online Supplementary Document
