# Supplementary material for: Prevalence and incidence of infections among people with type 2 diabetes: a systematic review and meta-analysis
Source: J Glob Health. 2026 Mar 27;16:04105. doi: 10.7189/jogh.16.04105 (PMC13021059; doi:10.7189/jogh.16.04105)
Supplement: Online Supplementary Document [file jogh-16-04105-s001.pdf]

Supplement to: Fu X, Chen W, Zhao H, Li M, Zhuang M, Xiao Y, Wu B, Guo J. Prevalence and incidence of infections among people with type 2 diabetes: a systematic review and meta-analysis. J Glob Health. 2026;16:04105.

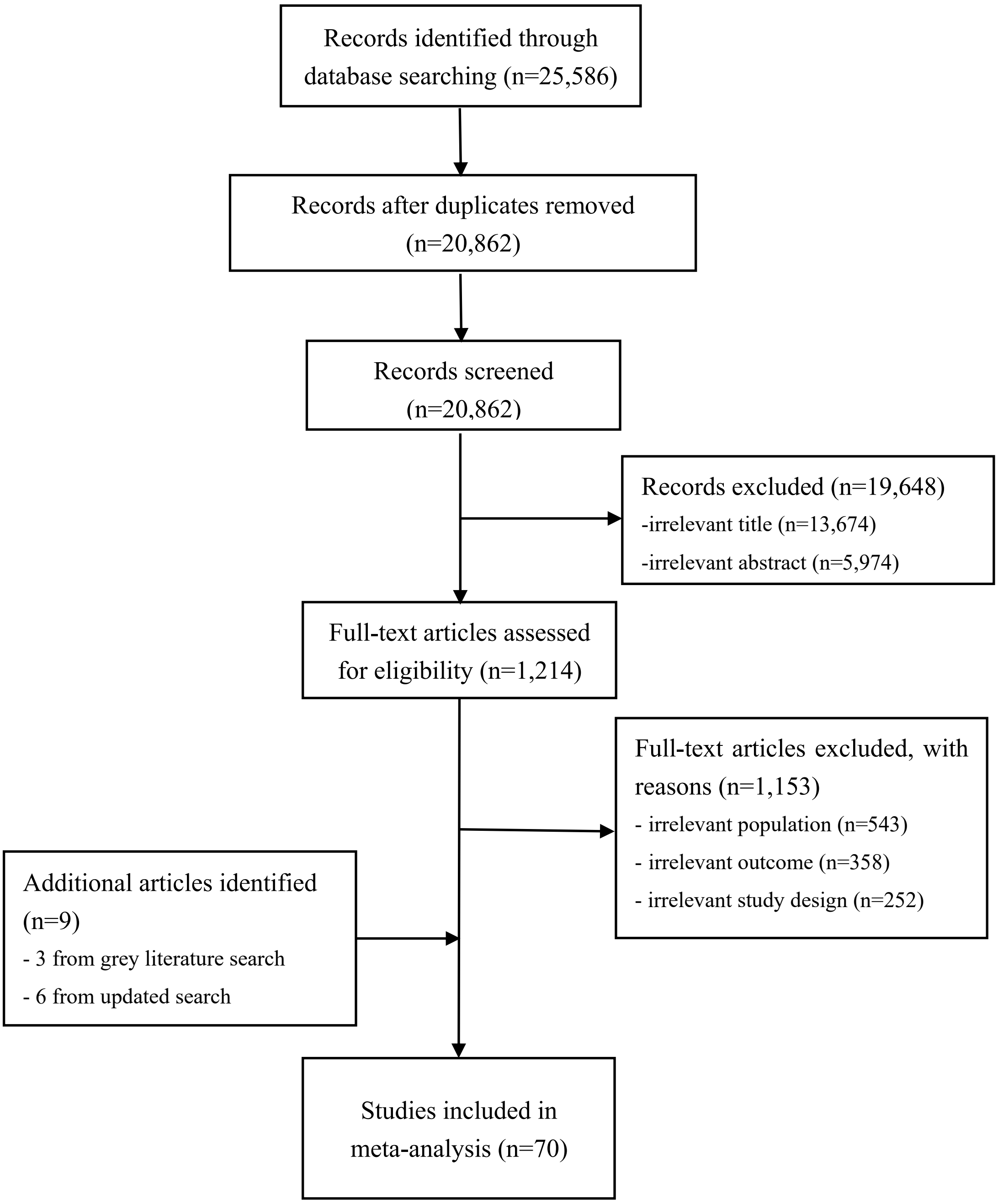

Supplemental Fig. 1 Flow diagram for identifying studies

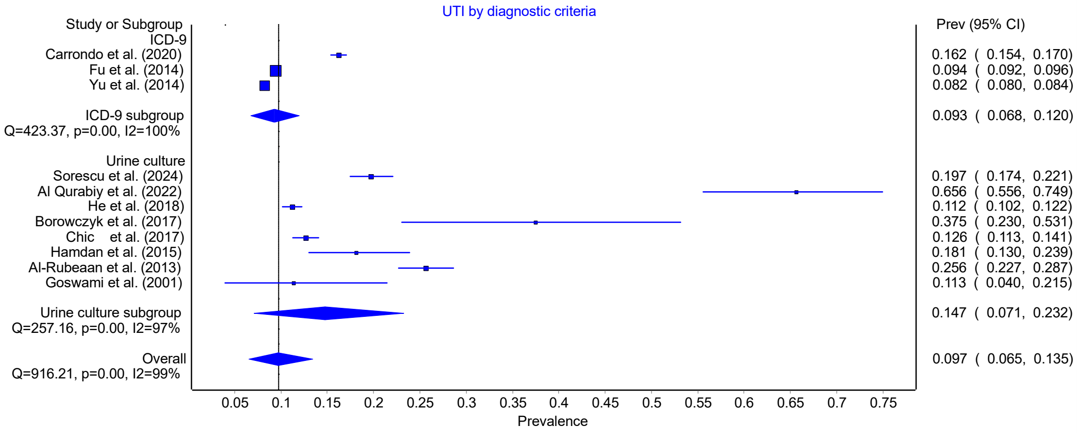

Supplemental Fig. 2 Forest plots of prevalence of urinary tract infections among people with type 2 diabetes by diagnostic criteria.

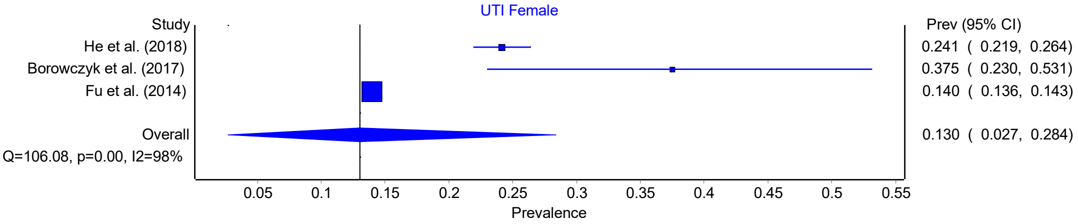

(A)

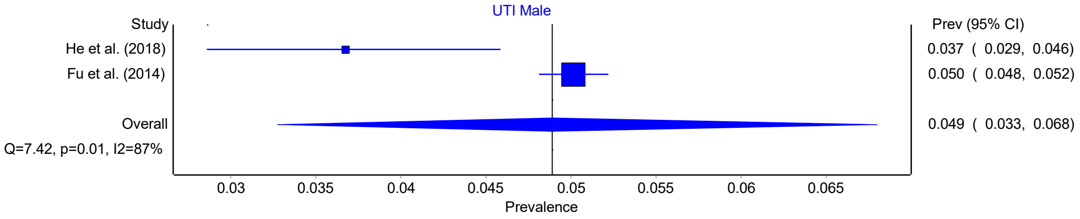

(B)

Supplemental Fig. 3 Forest plots of prevalence of urinary tract infections (A) among females and (B) among males with type 2 diabetes

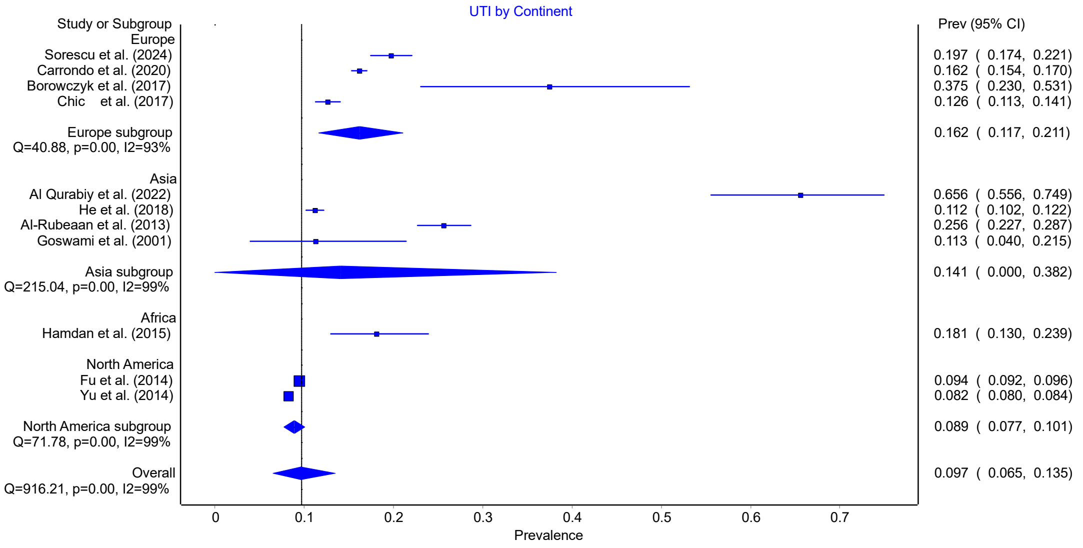

Supplemental Fig. 4 Forest plots of prevalence of urinary tract infections among people with type 2 diabetes by continents

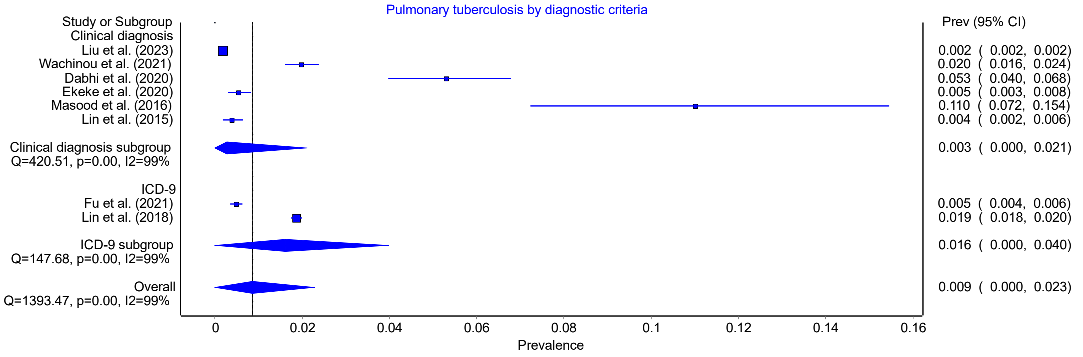

Supplemental Fig. 5 Forest plots of prevalence of pulmonary tuberculosis among people with type 2 diabetes by diagnostic criteria

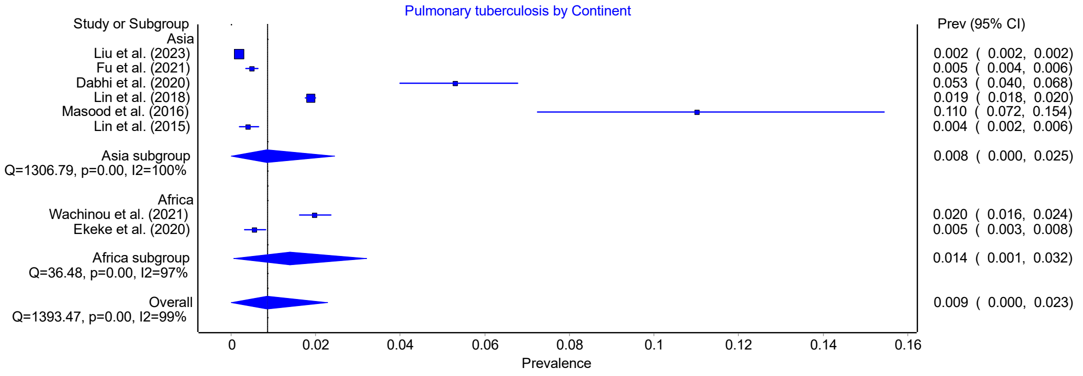

Supplemental Fig. 6 Forest plots of prevalence of pulmonary tuberculosis among people with type 2 diabetes by continents

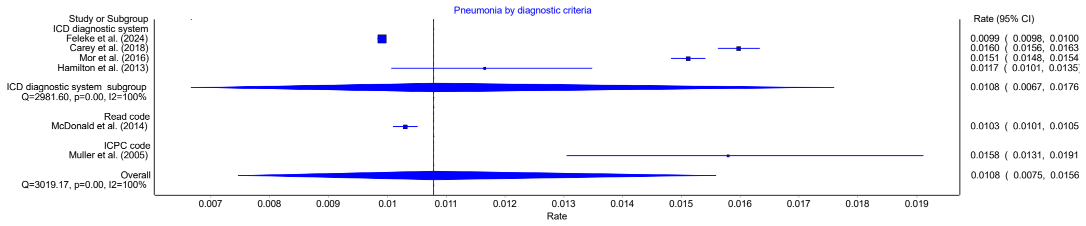

Supplemental Fig. 7 Forest plots of incidence of pneumonia among people with type 2 diabetes by diagnostic criteria.

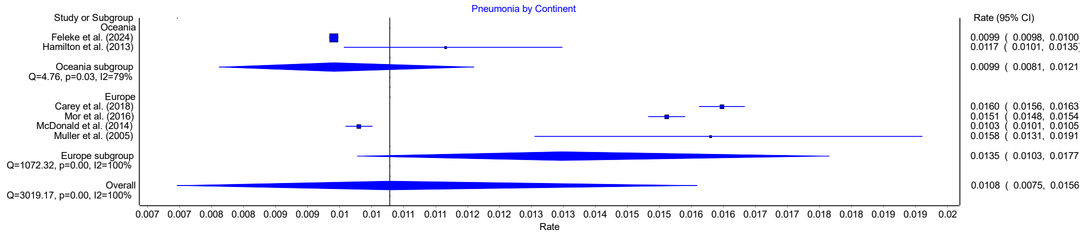

Supplemental Fig. 8 Forest plots of incidence of pneumonia among people with type 2 diabetes by continents.

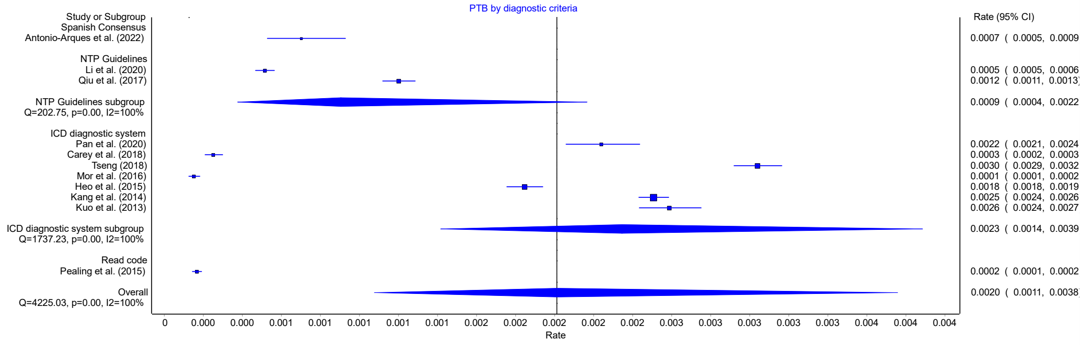

Supplemental Fig. 9 Forest plots of incidence of pulmonary tuberculosis among people with type 2 diabetes by diagnostic criteria.

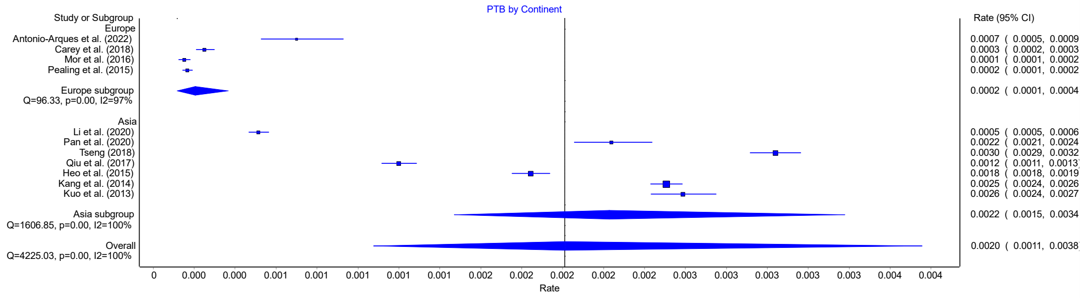

Supplemental Fig. 10 Forest plots of incidence of pulmonary tuberculosis among people with type 2 diabetes by continents

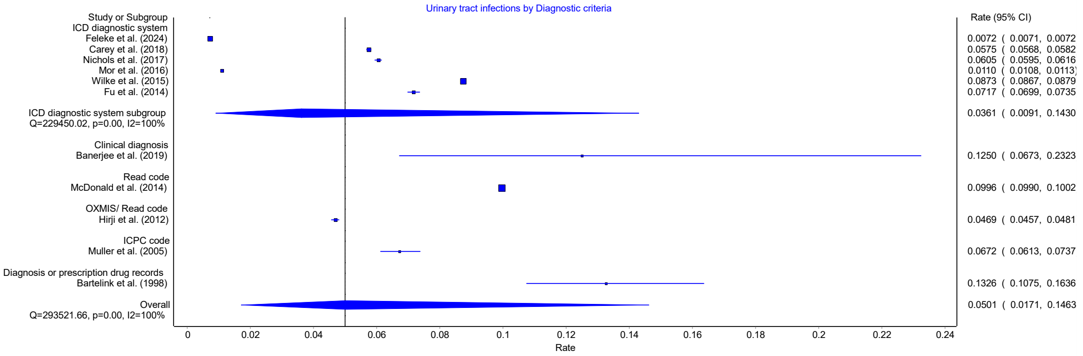

Supplemental Fig. 11 Forest plots of incidence of urinary tract infections among people with type 2 diabetes by diagnostic criteria.

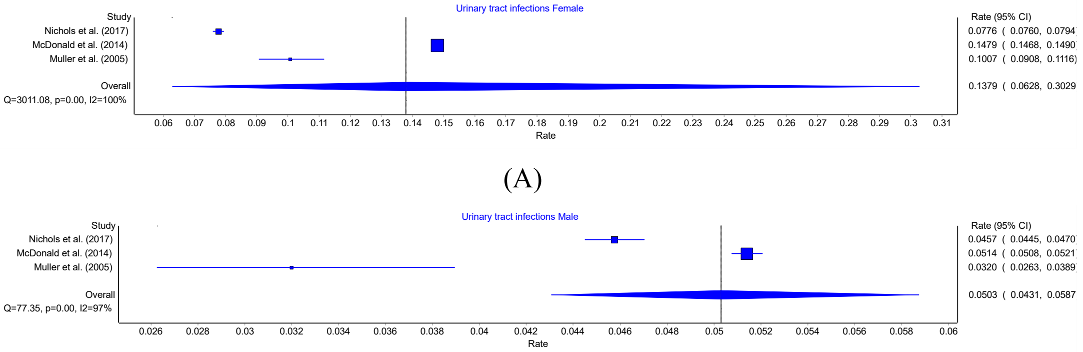

(B)

Supplemental Fig. 12 Forest plots of incidence of urinary tract infections (A) among females and (B) among males with type 2 diabetes

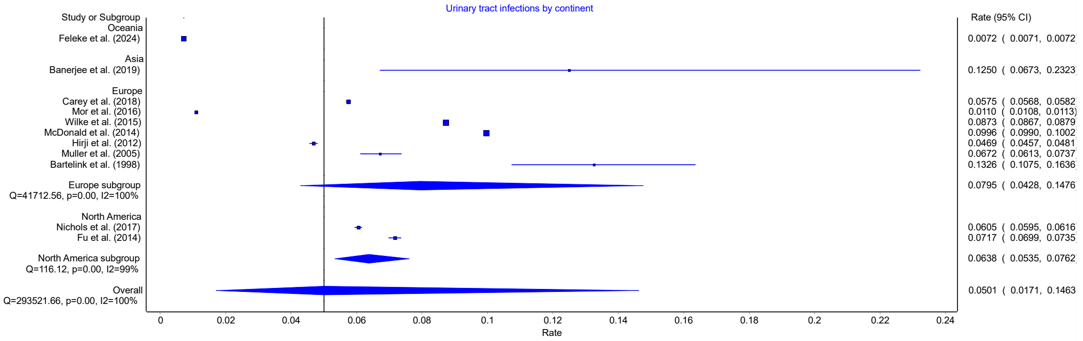

Supplemental Fig. 13 Forest plots of incidence of urinary tract infections among people with type 2 diabetes by continents

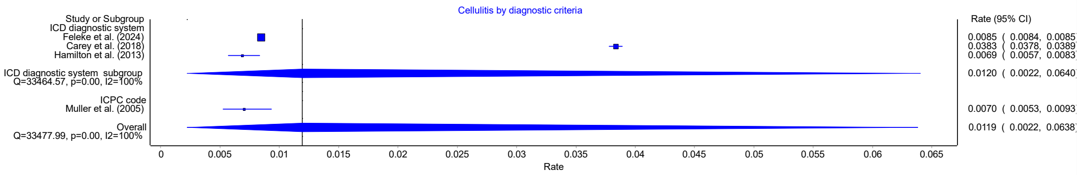

Supplemental Fig. 14 Forest plots of incidence of cellulitis among people with type 2 diabetes by diagnostic criteria.

Supplemental Table 1 Search strategy

PubMed

#1	"Infections"[MeSH Terms] OR "infectio*"[Title/Abstract] OR "Abscess"[MeSH Terms] OR "abscess*"[Title/Abstract] OR "Ecthyma"[MeSH Terms] OR "Ecthyma"[Title/Abstract] OR "Suppuration"[MeSH Terms] OR "suppurati*"[Title/Abstract] OR "Tracheitis"[MeSH Terms] OR "Tracheitis"[Title/Abstract] OR "Bronchitis"[MeSH Terms] OR "Bronchitis"[Title/Abstract] OR "Bronchiolitis"[MeSH Terms] OR "Bronchiolitis"[Title/Abstract] OR "Pneumonia"[MeSH Terms] OR "pneumonia*"[Title/Abstract] OR "Pneumonitis"[Title/Abstract] OR "Rhinoscleroma"[MeSH Terms] OR "Rhinoscleroma"[Title/Abstract] OR "Tuberculosis"[MeSH Terms] OR "tuberculos*"[Title/Abstract] OR "Whooping Cough"[MeSH Terms] OR "Whooping Cough"[Title/Abstract] OR "pertuss*"[Title/Abstract] OR "Empyema"[MeSH Terms] OR "Empyema"[Title/Abstract] OR "Cystitis"[MeSH Terms] OR "Cystitis"[Title/Abstract] OR "Pyelonephritis"[MeSH Terms] OR "Pyelonephritis"[Title/Abstract] OR "Urinary Tract Infections"[MeSH Terms] OR "Urinary Tract Infections"[Title/Abstract] OR "Gastroenteritis"[MeSH Terms] OR "Gastroenteritis"[Title/Abstract] OR "Hepatitis"[MeSH Terms] OR "Hepatitis"[Title/Abstract] OR "Cholecystitis"[MeSH Terms] OR "Cholecystitis"[Title/Abstract] OR "Appendicitis"[MeSH Terms] OR "Appendicitis"[Title/Abstract] OR "Peritonitis"[MeSH Terms] OR "Peritonitis"[Title/Abstract] OR "Typhlitis"[MeSH Terms] OR "Typhlitis"[Title/Abstract] OR "Cecitis"[Title/Abstract] OR "Osteomyelitis"[MeSH Terms] OR "Osteomyelitis"[Title/Abstract] OR "Discitis"[MeSH Terms] OR "Discitis"[Title/Abstract] OR "Diskitis"[Title/Abstract] OR "Myelitis"[MeSH Terms] OR "Myelitis"[Title/Abstract] OR "Encephalitis"[MeSH Terms] OR "Encephalitis"[Title/Abstract] OR "Encephalomyelitis"[MeSH Terms] OR "Encephalomyelitis"[Title/Abstract] OR "Meningitis"[MeSH Terms] OR "Meningitis"[Title/Abstract] OR "Pachymeningitis"[Title/Abstract] OR "Meningoencephalitis"[MeSH Terms] OR "Meningoencephalitis"[Title/Abstract] OR "Cerebromeningitis"[Title/Abstract] OR "Encephalomeningitis"[Title/Abstract] OR "Cerebral Ventriculitis"[MeSH Terms] OR "Ventriculitis"[Title/Abstract] OR "Mucormycosis"[MeSH Terms] OR "Mucormycosis"[Title/Abstract] OR "mucormycose*"[Title/Abstract] OR "Endocarditis"[MeSH Terms] OR "Endocarditis"[Title/Abstract] OR "Myocarditis"[MeSH Terms] OR "Myocarditis"[Title/Abstract] OR "Carditis"[Title/Abstract] OR "Sepsis"[MeSH Terms] OR "Sepsis"[Title/Abstract] OR "pyemia*"[Title/Abstract] OR "pyohemia*"[Title/Abstract] OR "pyaemia*"[Title/Abstract] OR "septicemia*"[Title/Abstract] OR "Bacteremia"[MeSH Terms] OR "bacteremia*"[Title/Abstract] OR "Toxemia"[MeSH Terms] OR "toxemia*"[Title/Abstract] OR "Endotoxemia"[MeSH Terms] OR "endotoxemia*"[Title/Abstract] OR "Viremia"[MeSH Terms] OR "viremia*"[Title/Abstract] OR "Cellulitis"[MeSH Terms] OR "Cellulitis"[Title/Abstract] OR "Phlegmon"[Title/Abstract] OR "fasciitis, necrotizing"[MeSH Terms] OR "Necrotizing Fasciitis"[Title/Abstract] OR "Necrotizing Fascitis"[Title/Abstract] OR "Lymphangitis"[MeSH Terms] OR

	"Lymphangitis"[Title/Abstract] OR "Lymphangitides"[Title/Abstract] OR "Lymphadenitis"[MeSH Terms] OR "Lymphadenitis"[Title/Abstract] OR "Adenitis"[Title/Abstract] OR "Soft Tissue Infections"[MeSH Terms] OR "Soft Tissue Infections"[Title/Abstract] OR "Paronychia"[MeSH Terms] OR "paronychia*"[Title/Abstract] OR "Otitis"[MeSH Terms] OR "Otitis"[Title/Abstract] OR "Mastoiditis"[MeSH Terms] OR "Mastoiditis"[Title/Abstract] OR "Sinusitis"[MeSH Terms] OR "Sinusitis"[Title/Abstract] OR "Supraglottitis"[MeSH Terms] OR "Supraglottitis"[Title/Abstract] OR "Laryngitis"[MeSH Terms] OR "Laryngitis"[Title/Abstract] OR "Laryngitides"[Title/Abstract] OR "Legionellosis"[MeSH Terms] OR "legionellos*"[Title/Abstract] OR "Epiglottitis"[MeSH Terms] OR "Epiglottitis"[Title/Abstract] OR "Pharyngitis"[MeSH Terms] OR "Pharyngitis"[Title/Abstract] OR "sore throat*"[Title/Abstract] OR "Pleurisy"[MeSH Terms] OR "Pleurisy"[Title/Abstract] OR "Pleurisies"[Title/Abstract] OR "Pleuritis"[Title/Abstract] OR "Conjunctivitis"[MeSH Terms] OR "Conjunctivitis"[Title/Abstract] OR "pink eye*"[Title/Abstract] OR "Corneal Ulcer"[MeSH Terms] OR "Corneal Ulcer"[Title/Abstract] OR "Ulcerative Keratitis"[Title/Abstract] OR "Endophthalmitis"[MeSH Terms] OR "Endophthalmitis"[Title/Abstract] OR "ophthalmia*"[Title/Abstract] OR "Periodontitis"[MeSH Terms] OR "Periodontitis"[Title/Abstract] OR "Periodontitides"[Title/Abstract] OR "Gingivitis"[MeSH Terms] OR "Gingivitis"[Title/Abstract] OR "Candidiasis"[MeSH Terms] OR "candidias*"[Title/Abstract] OR "monilias*"[Title/Abstract] OR "Erysipelas"[MeSH Terms] OR "Erysipelas"[Title/Abstract] OR ("Vaginitis"[Mesh Terms] OR Vaginitis[Title/Abstract] OR Vaginitides[Title/Abstract]) OR ("Balanitis"[Mesh Terms] OR Balanitis[Title/Abstract] OR Balanitides[Title/Abstract]) OR ("Tonsillitis"[Mesh Terms] OR Tonsillitis[Title/Abstract] OR Tonsillitides[Title/Abstract])
#2	"diabetes mellitus, type 2"[MeSH Terms] OR "T2D"[Title/Abstract] OR "T2DM"[Title/Abstract] OR "NIDDM"[Title/Abstract] OR (("non insulin"[Title/Abstract] OR "noninsulin"[Title/Abstract] OR "type 2"[Title/Abstract] OR "type II"[Title/Abstract]) AND ("diabetes"[Title/Abstract] OR "diabetic"[Title/Abstract] OR "diabetics"[Title/Abstract]))
#3	"Prevalence"[MeSH Terms] OR "Prevalence"[Title/Abstract] OR "Incidence"[MeSH Terms] OR "Incidence"[Title/Abstract] OR "Rate"[Title/Abstract]
#4	#1 AND #2 AND #3
#5	"COVID-19"[Title/Abstract] OR "2019 nCoV"[Title/Abstract] OR "SARS-CoV-2"[Title/Abstract] OR "2019 Novel Coronavirus"[Title/Abstract] OR "Severe Acute Respiratory Syndrome"[Title/Abstract] OR "SARS"[Title/Abstract] OR "Gestational Diabetes Mellitus"[Title/Abstract] OR "Gestational diabetes"[Title/Abstract] OR "diabetes gestational"[Title/Abstract] OR "Pregnancy-Induced Diabetes"[Title/Abstract]
#6	#4 NOT #5 Filters: Humans

Embase

#1	('infection'/exp OR infectio*:ab,ti) OR ('abscess'/exp OR abscess*:ab,ti) OR ('ecthyma'/exp OR ecthyma:ab,ti) OR ('suppuration'/exp OR suppurati*:ab,ti) OR ('tracheitis'/exp OR tracheitis:ab,ti) OR ('bronchitis'/exp OR bronchitis:ab,ti) OR ('bronchiolitis'/exp OR bronchiolitis:ab,ti) OR ('pneumonia'/exp OR pneumonia*:ab,ti OR pneumonitis:ab,ti) OR ('rhinoscleroma'/exp OR rhinoscleroma:ab,ti) OR ('tuberculosis'/exp OR tuberculos*:ab,ti) OR ('pertussis'/exp OR 'whooping cough':ab,ti OR pertuss*:ab,ti) OR ('empyema'/exp OR empyema:ab,ti) OR ('cystitis'/exp OR cystitis:ab,ti) OR ('pyelonephritis'/exp OR pyelonephritis:ab,ti) OR ('urinary tract infection'/exp OR 'urinary tract infection':ab,ti) OR ('gastroenteritis'/exp OR gastroenteritis:ab,ti) OR ('hepatitis'/exp OR hepatitis:ab,ti) OR ('cholecystitis'/exp OR cholecystitis:ab,ti) OR ('appendicitis'/exp OR appendicitis:ab,ti) OR ('peritonitis'/exp OR peritonitis:ab,ti) OR ('typhlitis'/exp OR typhlitis:ab,ti OR cecitis:ab,ti) OR ('osteomyelitis'/exp OR osteomyelitis:ab,ti) OR ('diskitis'/exp OR discitis:ab,ti OR diskitis:ab,ti) OR ('myelitis'/exp OR myelitis:ab,ti) OR ('encephalitis'/exp OR encephalitis:ab,ti) OR ('encephalomyelitis'/exp OR encephalomyelitis:ab,ti) OR ('meningitis'/exp OR meningitis:ab,ti OR pachymeningitis:ab,ti) OR ('meningoencephalitis'/exp OR meningoencephalitis:ab,ti OR cerebromeningitis:ab,ti OR encephalomeningitis:ab,ti) OR ('brain ventriculitis'/exp OR ventriculitis:ab,ti) OR ('mucormycosis'/exp OR mucormycosis:ab,ti OR mucormycose*:ab,ti) OR ('endocarditis'/exp OR endocarditis:ab,ti) OR ('myocarditis'/exp OR myocarditis:ab,ti OR carditis:ab,ti) OR ('sepsis'/exp OR sepsis:ab,ti OR pyemia*:ab,ti OR pyohemia*:ab,ti OR pyaemia*:ab,ti OR septicemia*:ab,ti) OR ('bacteremia'/exp OR bacteremia*:ab,ti) OR ('toxemia'/exp OR toxemia*:ab,ti) OR ('endotoxemia'/exp OR endotoxemia*:ab,ti) OR ('viremia'/exp OR viremia*:ab,ti) OR ('cellulitis'/exp OR cellulitis:ab,ti OR phlegmon:ab,ti) OR ('necrotizing fasciitis'/exp OR 'necrotizing fasciitis':ab,ti OR 'necrotizing fascitis':ab,ti) OR ('lymphangitis'/exp OR lymphangitis:ab,ti OR lymphangitides:ab,ti) OR ('lymphadenitis'/exp OR lymphadenitis:ab,ti OR adenitis:ab,ti) OR ('paronychia'/exp OR paronychia*:ab,ti) OR ('otitis'/exp OR otitis:ab,ti) OR ('mastoiditis'/exp OR mastoiditis:ab,ti) OR ('sinusitis'/exp OR sinusitis:ab,ti) OR (‘supraglottitis’/exp OR supraglottitis:ab,ti) OR ('laryngitis'/exp OR laryngitis:ab,ti OR laryngitides:ab,ti) OR ('legionellosis'/exp OR legionellos*:ab,ti) OR ('epiglottitis'/exp OR epiglottitis:ab,ti) OR ('pharyngitis'/exp OR pharyngitis:ab,ti OR 'sore throat*':ab,ti) OR ('pleurisy'/exp OR pleurisy:ab,ti OR pleurisies:ab,ti OR pleuritis:ab,ti) OR ('conjunctivitis'/exp OR conjunctivitis:ab,ti OR 'pink eye*':ab,ti) OR ('cornea ulcer'/exp OR 'cornea ulcer':ab,ti OR 'ulcerative keratitis':ab,ti) OR ('endophthalmitis'/exp OR endophthalmitis:ab,ti OR ophthalmia*:ab,ti) OR ('periodontitis'/exp OR periodontitis:ab,ti OR periodontitides:ab,ti) OR ('gingivitis'/exp OR gingivitis:ab,ti) OR ('candidiasis'/exp OR candidias*:ab,ti OR monilias*:ab,ti) OR ('erysipelas'/exp OR erysipelas:ab,ti) OR (Vaginitis'/exp OR Vaginitis:ab,ti OR Vaginitides:ab,ti) OR (Balanitis'/exp OR Balanitis:ab,ti OR Balanitides:ab,ti) OR (Tonsillitis'/exp OR Tonsillitis:ab,ti OR Tonsillitides:ab,ti)
#2	'non insulin dependent diabetes mellitus'/exp OR t2d:ab,ti OR t2dm:ab,ti OR niddm:ab,ti OR (('non insulin':ab,ti OR noninsulin:ab,ti OR 'type 2':ab,ti OR 'type II':ab,ti) AND (diabetes:ab,ti

	OR diabetic:ab,ti OR diabetics:ab,ti))
#3	('prevalence'/exp OR prevalence:ab,ti) OR ('incidence'/exp OR incidence:ab,ti) OR rate:ab,ti
#4	#1 AND #2 AND #3
#5	'covid 19':ab,ti OR '2019 ncov':ab,ti OR 'sars-cov-2':ab,ti OR '2019 novel coronavirus':ab,ti OR 'severe acute respiratory syndrome':ab,ti OR sars:ab,ti OR 'gestational diabetes mellitus':ab,ti OR 'gestational diabetes':ab,ti OR 'diabetes, gestational':ab,ti OR 'pregnancy-induced diabetes':ab,ti
#6	#4 NOT #5 AND [humans]/lim

Web of Science

#1	TS=(Infectio* OR Abscess* OR Ecthyma OR Suppurati* OR Tracheitis OR Bronchitis OR Bronchiolitis OR Pneumonia* OR Pneumonitis OR Rhinoscleroma OR Tuberculos* OR "Whooping Cough" OR Pertuss* OR Empyema OR Cystitis OR Pyelonephritis OR "Urinary Tract Infections" Gastroenteritis OR Hepatitis OR Cholecystitis OR Appendicitis OR Peritonitis OR Typhlitis OR Cecitis OR Osteomyelitis OR Discitis OR Diskitis OR Myelitis OR Encephalitis OR Encephalomyelitis OR Meningitis OR Pachymeningitis OR Meningoencephalitis OR Cerebromeningitis OR Encephalomeningitis OR "Ventriculitis" OR Mucormycosis OR Mucormycose* OR Endocarditis OR Myocarditis OR Carditis OR Sepsis OR Pyemia* OR Pyohemia* OR Pyaemia* OR Septicemia* OR Bacteremia* OR Toxemia* OR Endotoxemia* OR Viremia* OR Cellulitis OR Phlegmon OR "Fasciitis, Necrotizing" OR "Necrotizing Fasciitis" OR "Necrotizing Fascitis" OR Lymphangitis OR Lymphangitides OR Lymphadenitis OR Adenitis OR Paronychia* OR Otitis OR Mastoiditis OR Sinusitis OR Supraglottitis OR Laryngitis OR Laryngitides OR Legionellos* OR Epiglottitis OR Pharyngitis OR "Sore Throat*" OR Pleurisy OR Pleurisies OR Pleuritis OR Conjunctivitis OR "Pink Eye*" OR "Corneal Ulcer" OR "Ulcerative Keratitis" OR Endophthalmitis OR Ophthalmia* OR Periodontitis OR Periodontitides OR Gingivitis OR Candidias* OR Monilias* OR Erysipelas) OR (Vaginitis OR Vaginitides[Title/Abstract]) OR (Balanitis OR Balanitides) OR (Tonsillitis OR Tonsillitides)
#2	TS=(T2D OR T2DM OR NIDDM OR ((non insulin OR noninsulin OR type 2 OR type II) AND (diabetes OR diabetic OR diabetics)))
#3	TS=(Prevalence OR Incidence OR Rate)
#4	#1 AND #2 AND #3
#5	TS=("COVID-19" OR "2019 nCoV" OR "SARS-CoV-2" OR "2019 Novel Coronavirus" OR "Severe Acute Respiratory Syndrome" OR SARS OR "Gestational Diabetes Mellitus" OR "Gestational diabetes" OR "Diabetes, Gestational" OR "Pregnancy-Induced Diabetes")
#6	#4 NOT #5

Cochrane Library

#1	MeSH descriptor: [Infections] explode all trees
#2	MeSH descriptor: [Abscess] explode all trees
#3	MeSH descriptor: [Ecthyma] explode all trees
#4	MeSH descriptor: [Suppuration] explode all trees
#5	MeSH descriptor: [Tracheitis] explode all trees
#6	MeSH descriptor: [Bronchitis] explode all trees
#7	MeSH descriptor: [Bronchiolitis] explode all trees
#8	MeSH descriptor: [Pneumonia] explode all trees
#9	MeSH descriptor: [Rhinoscleroma] explode all trees
#10	MeSH descriptor: [Tuberculosis] explode all trees
#11	MeSH descriptor: [Whooping Cough] explode all trees
#12	MeSH descriptor: [Empyema] explode all trees
#13	MeSH descriptor: [Cystitis] explode all trees
#14	MeSH descriptor: [Pyelonephritis] explode all trees
#15	MeSH descriptor: [Urinary Tract Infections] explode all trees
#16	MeSH descriptor: [Gastroenteritis] explode all trees
#17	MeSH descriptor: [Hepatitis] explode all trees
#18	MeSH descriptor: [Cholecystitis] explode all trees
#19	MeSH descriptor: [Appendicitis] explode all trees
#20	MeSH descriptor: [Peritonitis] explode all trees
#21	MeSH descriptor: [Typhlitis] explode all trees
#22	MeSH descriptor: [Osteomyelitis] explode all trees
#23	MeSH descriptor: [Discitis] explode all trees
#24	MeSH descriptor: [Myelitis] explode all trees
#25	MeSH descriptor: [Encephalitis] explode all trees
#26	MeSH descriptor: [Encephalomyelitis] explode all trees
#27	MeSH descriptor: [Meninges] explode all trees
#28	MeSH descriptor: [Meningoencephalitis] explode all trees
#29	MeSH descriptor: [Cerebral Ventriculitis] explode all trees
#30	MeSH descriptor: [Mucormycosis] explode all trees
#31	MeSH descriptor: [Endocarditis] explode all trees
#32	MeSH descriptor: [Myocarditis] explode all trees
#33	MeSH descriptor: [Sepsis] explode all trees
#34	MeSH descriptor: [Bacteremia] explode all trees
#35	MeSH descriptor: [Toxemia] explode all trees
#36	MeSH descriptor: [Endotoxemia] explode all trees
#37	MeSH descriptor: [Viremia] explode all trees

#38	MeSH descriptor: [Cellulitis] explode all trees
#39	MeSH descriptor: [Fasciitis, Necrotizing] explode all trees
#40	MeSH descriptor: [Lymphangitis] explode all trees
#41	MeSH descriptor: [Lymphadenitis] explode all trees
#42	MeSH descriptor: [Soft Tissue Infections] explode all trees
#43	MeSH descriptor: [Paronychia] explode all trees
#44	MeSH descriptor: [Otitis] explode all trees
#45	MeSH descriptor: [Mastoiditis] explode all trees
#46	MeSH descriptor: [Sinusitis] explode all trees
#47	MeSH descriptor: [Supraglottitis] explode all trees
#48	MeSH descriptor: [Laryngitis] explode all trees
#49	MeSH descriptor: [Legionellosis] explode all trees
#50	MeSH descriptor: [Epiglottitis] explode all trees
#51	MeSH descriptor: [Pharyngitis] explode all trees
#52	MeSH descriptor: [Pleurisy] explode all trees
#53	MeSH descriptor: [Conjunctivitis] explode all trees
#54	MeSH descriptor: [Corneal Ulcer] explode all trees
#55	MeSH descriptor: [Endophthalmitis] explode all trees
#56	MeSH descriptor: [Periodontitis] explode all trees
#57	MeSH descriptor: [Gingivitis] explode all trees
#58	MeSH descriptor: [Candidiasis] explode all trees
#59	MeSH descriptor: [Erysipelas] explode all trees
#60	MeSH descriptor: [Vaginitis] explode all trees
#61	MeSH descriptor: [Balanitis] explode all trees
#62	MeSH descriptor: [Tonsillitis] explode all trees
#63	#1 OR #2 OR #3 OR #4 OR #5 OR #6 OR #7 OR #8 OR #9 OR #10 OR #11 OR #12 OR #13 OR #14 OR #15 OR #16 OR #17 OR #18 OR #19 OR #20 OR #21 OR #22 OR #23 OR #24 OR #25 OR #26 OR #27 OR #28 OR #29 OR #30 OR #31 OR #32 OR #33 OR #34 OR #35 OR #36 OR #37 OR #38 OR #39 OR #40 OR #41 OR #42 OR #43 OR #44 OR #45 OR #46 OR #47 OR #48 OR #49 OR #50 OR #51 OR #52 OR #53 OR #54 OR #55 OR #56 OR #57 OR #58 OR #59 OR #60 OR #61 OR #62
#64	(Infectio* OR Abscess* OR Ecthyma OR Suppurati* OR Tracheitis OR Bronchitis OR Bronchiolitis OR Pneumonia* OR Pneumonitis OR Rhinoscleroma OR Tuberculos* OR "Whooping Cough" OR Pertuss* OR Empyema OR Cystitis OR Pyelonephritis OR "Urinary Tract Infections" Gastroenteritis OR Hepatitis OR Cholecystitis OR Appendicitis OR Peritonitis OR Typhlitis OR Cecitis OR Osteomyelitis OR Discitis OR Diskitis OR Myelitis OR Encephalitis OR Encephalomyelitis OR Meningitis OR Pachymeningitis OR Meningoencephalitis OR Cerebromeningitis OR Encephalomeningitis OR "Ventriculitis" OR Mucormycosis OR Mucormycose* OR Endocarditis OR Myocarditis OR Carditis OR

	Sepsis OR Pyemia* OR Pyohemia* OR Pyaemia* OR Septicemia* OR Bacteremia* OR Toxemia* OR Endotoxemia* OR Viremia* OR Cellulitis OR Phlegmon OR "Fasciitis, Necrotizing" OR "Necrotizing Fasciitis" OR "Necrotizing Fascitis" OR Gangrene* OR Lymphangitis OR Lymphangitides OR Lymphadenitis OR Adenitis OR Paronychia* OR Otitis OR Mastoiditis OR Sinusitis OR Supraglottitis OR Laryngitis OR Laryngitides OR Legionellos* OR Epiglottitis OR Pharyngitis OR (Sore NEXT Throat*) OR Pleurisy OR Pleurisies OR Pleuritis OR Conjunctivitis OR (Pink NEXT Eye*) OR "Corneal Ulcer" OR "Ulcerative Keratitis" OR Endophthalmitis OR Ophthalmia* OR Periodontitis OR Periodontitides OR Gingivitis OR Candidias* OR Monilias* OR Erysipelas):ti,ab,kw
#65	#63 OR #64
#66	MeSH descriptor: [Diabetes Mellitus, Type 2] explode all trees
#67	(T2D OR T2DM OR NIDDM):ti,ab,kw OR ((non insulin OR noninsulin OR type 2 OR type II):ti,ab,kw AND (diabetes OR diabetic OR diabetics):ti,ab,kw)
#68	#66 OR #67
#69	MeSH descriptor: [Prevalence] explode all trees
#70	MeSH descriptor: [Incidence] explode all trees
#71	(prevalence OR incidence OR rate):ti,ab,kw
#72	#69 OR #70 OR #71
#73	(COVID-19 OR "2019 nCoV" OR "SARS-CoV-2" OR "2019 Novel Coronavirus" OR "Severe Acute Respiratory Syndrome" OR SARS OR "Gestational Diabetes Mellitus" OR "Gestational diabetes" OR "Diabetes, Gestational" OR "Pregnancy-Induced Diabetes"):ti,ab,kw
#74	#65 AND #68 AND #72 NOT #73

Supplemental Table 2 Quality assessment for prevalence studies

Author, year	Q1	Q2	Q3	Q4	Q5	Q6	Q7	Q8	Q9	Total	Quality
Sorescu et al., 2024 [63]	N	N	U	Y	Y	Y	Y	Y	NA	5	Moderate
Liu et al., 2023 [62]	N	N	Y	Y	Y	Y	Y	Y	U	6	Moderate
Todescan et al., 2023 [59]	N	N	U	Y	Y	Y	Y	Y	Y	6	Moderate
Al Qurabiy et al., 2022 [31]	N	N	U	Y	Y	Y	Y	N	U	4	Low
Fu et al., 2021 [40]	N	N	Y	Y	Y	Y	Y	Y	U	6	Moderate
Wachinou et al., 2021 [61]	N	N	Y	Y	Y	Y	Y	Y	U	6	Moderate
Carrondo et al., 2020 [34]	Y	Y	Y	Y	Y	Y	Y	Y	U	8	High
Dabhi et al., 2020 [36]	N	N	Y	Y	Y	Y	Y	N	U	5	Moderate
Ekeke et al., 2020 [37]	N	N	Y	Y	Y	Y	Y	N	U	5	Moderate
Rasid et al., 2020 [53]	N	Y	Y	Y	Y	Y	Y	Y	U	7	High
Saleh et al., 2020 [55]	N	Y	Y	Y	Y	Y	Y	Y	NA	7	High
He et al., 2018 [43]	N	Y	Y	Y	N	Y	Y	Y	U	6	Moderate
Lin et al., 2018 [12]	Y	Y	Y	Y	Y	Y	Y	Y	U	8	High
Borowczyk et al., 2017 [33]	N	N	U	Y	Y	Y	Y	Y	U	5	Moderate
Nitta et al., 2017 [49]	N	N	Y	Y	Y	Y	Y	Y	U	6	Moderate
Pham et al., 2018 [50]	N	N	Y	Y	Y	Y	Y	Y	U	6	Moderate
Pumerantz et al., 2017 [52]	N	N	Y	Y	Y	Y	Y	Y	U	6	Moderate
Chiţă et al., 2017 [35]	N	N	Y	Y	Y	Y	Y	Y	U	6	Moderate
Masood et al., 2016 [48]	N	N	Y	Y	Y	Y	Y	N	U	5	Moderate
Hamdan et al., 2015 [42]	N	N	U	Y	Y	Y	Y	Y	U	5	Moderate
Lin et al., 2015 [45]	N	N	Y	Y	Y	Y	Y	Y	U	6	Moderate
Fu et al., 2014 [39]	Y	Y	Y	Y	Y	Y	Y	Y	U	8	High
Masi et al., 2014 [47]	N	N	Y	Y	Y	Y	Y	Y	U	6	Moderate
Yu et al., 2014 [11]	N	Y	Y	Y	Y	Y	Y	Y	U	7	High
Al-Rubeaan et al., 2013 [32]	N	N	Y	Y	Y	Y	Y	Y	U	6	Moderate
Susanto et al., 2011 [58]	N	N	Y	Y	Y	Y	Y	Y	U	6	Moderate
Preshaw et al., 2010 [51]	N	N	Y	Y	Y	Y	Y	Y	U	6	Moderate
Silva et al., 2010 [57]	Y	Y	Y	Y	Y	Y	Y	Y	Y	9	High
Ahmed et al., 2009 [30]	N	N	U	Y	U	N	N	N	U	1	Low
Fernandes et al., 2009 [38]	N	N	U	Y	Y	Y	Y	Y	U	5	Moderate
Hintao et al., 2007 [44]	N	Y	Y	Y	Y	Y	Y	Y	Y	8	High
Malazy et al., 2007 [46]	N	N	U	Y	Y	Y	Y	N	U	4	Low
Movahed et al., 2007 [13]	N	Y	Y	Y	Y	Y	Y	Y	NA	7	High
Sasmaz et al., 2004 [56]	N	N	U	Y	Y	Y	Y	N	U	4	Low
Tsai et al., 2002 [60]	Y	Y	Y	Y	Y	Y	Y	Y	Y	9	High

Goswami et al., 2001 [41]	N	N	U	Y	Y	Y	Y	N	U	4	Low
Romano et al., 1998 [54]	N	N	Y	Y	Y	Y	Y	N	U	5	Moderate

Y=Yes; N=No; U=Unclear; NA=Not applicable

Supplemental Table 3 Quality assessment for incidence studies

Author, year	Q1	Q2	Q3	Q4	Q5	Q6	Q7	Q8	Q9	Total	Quality
Kim et al., 2025 [77]	Y	Y	Y	Y	Y	Y	Y	Y	NA	8	High
Lee et al., 2025 [80]	Y	Y	Y	Y	Y	Y	Y	Y	NA	8	High
Feleke et al., 2024 [69]	Y	Y	Y	Y	Y	Y	Y	Y	NA	8	High
Chien et al., 2023 [67]	Y	Y	Y	Y	Y	Y	Y	Y	NA	8	High
Antonio-Arques et al., 2022 [64]	N	Y	Y	Y	Y	Y	Y	Y	U	7	High
Lopez-de-Andres et al., 2022 [82]	Y	Y	Y	Y	Y	Y	Y	Y	NA	8	High
Poirrier et al., 2022 [89]	Y	Y	Y	Y	Y	Y	Y	Y	NA	8	High
Wang et al., 2021 [93]	Y	Y	Y	Y	Y	Y	Y	Y	NA	8	High
Wu et al., 2021 [95]	Y	Y	Y	Y	Y	Y	Y	Y	NA	8	High
Li et al., 2020 [81]	N	Y	Y	Y	Y	Y	Y	Y	NA	7	High
Pan et al., 2020 [87]	Y	Y	Y	Y	Y	Y	Y	Y	NA	8	High
Banerjee et al., 2019 [65]	N	Y	Y	Y	Y	Y	Y	N	U	6	Moderate
de Miguel-Yanes et al., 2019 [68]	Y	Y	Y	Y	Y	Y	Y	Y	NA	8	High
Ko et al., 2019 [78]	Y	Y	Y	Y	Y	Y	Y	Y	NA	8	High
Carey et al., 2018 [14]	Y	Y	Y	Y	Y	Y	Y	Y	U	8	High
Ferreira et al., 2018 [70]	Y	Y	Y	Y	Y	Y	Y	Y	NA	8	High
Tseng, 2018 [91]	Y	Y	Y	Y	Y	Y	Y	Y	NA	8	High
Wang et al., 2018 [92]	Y	Y	Y	Y	Y	Y	Y	Y	NA	8	High
Nichols et al., 2017 [86]	Y	Y	Y	Y	Y	Y	Y	Y	NA	8	High
Qiu et al., 2017 [90]	Y	Y	Y	Y	Y	Y	Y	Y	NA	8	High
Mor et al., 2016 [84]	Y	Y	Y	Y	Y	Y	Y	Y	NA	8	High
Heo et al., 2015 [73]	Y	Y	Y	Y	Y	Y	Y	Y	NA	8	High
Pealing et al., 2015 [88]	Y	Y	Y	Y	Y	Y	Y	Y	NA	8	Moderate
Wilke et al., 2015 [94]	Y	Y	Y	Y	Y	Y	Y	Y	NA	8	Moderate
Fu et al., 2014 [39]	Y	Y	Y	Y	Y	Y	Y	Y	U	8	High
Guignard et al., 2014 [71]	N	Y	Y	Y	Y	Y	Y	Y	NA	7	High
Kang et al., 2014 [76]	Y	Y	Y	Y	Y	Y	Y	Y	NA	8	High
McDonald et al., 2014 [83]	Y	Y	Y	Y	Y	Y	Y	Y	NA	8	High
Hamilton et al., 2013 [72]	N	Y	Y	Y	Y	Y	Y	Y	Y	8	High
Kuo et al., 2013 [79]	Y	Y	Y	Y	Y	Y	Y	Y	NA	8	High
Hirji et al., 2012 [74]	Y	Y	Y	Y	Y	Y	Y	Y	NA	7	High
Hirji et al., 2012 [75]	N	Y	Y	Y	Y	Y	Y	Y	NA	7	High
Muller et al., 2005 [85]	Y	Y	Y	Y	Y	Y	Y	Y	NA	8	High
Bartelink et al., 1998 [66]	N	Y	Y	Y	Y	Y	Y	Y	U	7	High

Y=Yes; N=No; U=Unclear; NA=Not applicable
